# miR-378a-5p exerts tumor-suppressive effects on esophageal squamous cell carcinoma after neoadjuvant immunotherapy by downregulating APOC1/CEP55

**DOI:** 10.1038/s41598-023-50938-z

**Published:** 2024-01-03

**Authors:** Yang Pengjie, Jia Rong, Ning Pengfei

**Affiliations:** 1 Inner Mongolia Medical University, Jinshan Development Zone, Hohhot, 010110 Inner Mongolia Autonomous Region China; 2 Thoracic Surgery Department, Peking University Cancer Hospital Inner Mongolia Hospital (Cancer Hospital Affiliated to Inner Mongolia Medical University), Hohhot, 010110 Inner Mongolia Autonomous Region China

**Keywords:** Computational biology and bioinformatics, Prognostic markers, Surgical oncology

## Abstract

Genetic assessment of tumors following neoadjuvant immunotherapy helps identifying targets that mediate anti-tumor immunity. In this study, we explored dysregulated RNAs in esophageal squamous cell carcinoma samples after neoadjuvant immunotherapy using deep sequencing and high-throughput screening. We identified 584 differentially expressed messenger RNAs (mRNAs), 67 differentially expressed microRNAs (miRNAs), and 1,047 differentially expressed long non-coding RNAs (lncRNAs) using differential expression analysis. Competing endogenous RNAs closely related to esophageal squamous cell carcinoma were selected via a combined Pearson’s correlation test and weighted correlation network analysis. After validation using survival analysis and dry-lab and wet-lab-based studies, we identified the I-miR-378-5p-APOC1/CEP55 as a critical pathway for esophageal squamous cell carcinoma progression after neoadjuvant immunotherapy. Tumor immune infiltration analysis showed that APOC1 and CEP55 expression is associated with immune regulatory pathways and the function of multiple infiltrating immune cells. We investigated the mechanism of esophageal squamous carcinoma progression after neoadjuvant immunotherapy from the perspective of the mRNA–miRNA–lncRNA network. Furthermore, we identified accurate novel therapeutic targets and prognostic biomarkers, introduced novel perspectives to immunotherapy studies, and laid the foundation for the clinical treatment of patients with esophageal squamous carcinoma.

## Introduction

Esophageal cancer is the sixth most common cancer globally^1^. It is an aggressive cancer type, and patients with esophageal cancer have poor prognosis. Esophageal cancer does not show obvious early signs, and most patients are diagnosed in the mid-late stages. It has a high malignancy rate, with a five-year survival rate of 15 to 25%. Surgical resection is the primary treatment method for esophageal cancer; however, the prognosis tends to be poor when surgery is the only treatment approach. In recent years, as a preoperative treatment plan, neoadjuvant immunotherapy has improved the local control of tumors and increased the possibility of surgery and the R0 resection, benefiting overall patient survival. Among these, the treatment modes of neoadjuvant immunotherapy and neoadjuvant radiotherapy have widely been used^2–4^. However, the best plan for neoadjuvant immunotherapy for esophageal cancer is still under debate, as studies have mainly focused on improving the patients’ prognosis and prolonging their survival rate.

Tumor immunotherapy controls and kills tumor cells while repairing and enhancing the immune system^5,6^. New strategies have been researched and explored, which has been accompanied by an improved understanding of the body’s anti-tumor immune response, tumor immune escape mechanisms, and the tumor microenvironment. Clinical research has demonstrated that immunotherapy is effective in patients with esophageal squamous cell carcinoma. Patients receiving immunotherapy have improved overall survival, disease-free survival, complete remission, partial remission, and overall efficacy rates^7^. Many experiments have been focused on the clinical level, in which the molecular biological mechanism of immunotherapy, however, still needs to be clarified. Therefore, discussing neoadjuvant immunotherapy for treating esophageal squamous cell carcinoma may help identify a potential population for efficacy evaluation after immunotherapy treatment.

In recent years, numerous studies have shown that non-coding RNAs, especially microRNAs (miRNAs) and long non-coding RNAs (lncRNAs), participate in many biological processes in tumor cells, such as development, differentiation, proliferation, and apoptosis, by altering the genetic code^8–10^. miRNAs mainly inhibit the translation of target messenger RNA (mRNA) or directly degrade the target mRNA by complementary pairing with the target mRNA at the post-transcriptional level^11–14^. lncRNAs can interact with mRNAs, miRNAs, and other lncRNAs and participate in multiple biological processes, including cell differentiation, cancer proliferation, and metastasis^15–18^. The competing endogenous RNA (ceRNA) network can compete for the same miRNA response elements (MREs)^19,20^, forming potential regulatory pathways such as mRNA–miRNA–lncRNA. The ceRNA regulatory network is more delicate and complicated and involves more RNA factors than miRNA and lncRNA networks. Nevertheless, it can comprehensively explain the onset and progression of cancer and provide a new perspective on transcriptome research related to the early identification, diagnosis, and treatment of cancer^21,22^. MiRNAs are widely involved in the metabolism, proliferation, migration, angiogenesis, and other processes of esophageal cancer. Chen et al.^23^ found in their study on the mechanism of radiation resistance in esophageal cancer that upregulation of MiR-20b-5p promotes cell proliferation, migration, invasion, and EMT processes, and reduces cell apoptosis through negative regulation of PTEN. MiR-125a-5p inhibits cell proliferation, migration, invasion, EMT processes, and induces cell apoptosis by negatively regulating IL6R. These data indicate that miR-20b-5p and miR-125a-5p promote tumorigenesis in radiation-resistant KYSE-150R cells and may serve as new targets for the treatment of esophageal cancer. He et al.^24^ showed that overexpression of miR-203 significantly reduced the proliferation, migration, and invasion of esophageal squamous cell carcinoma cells. Bioinformatics analysis showed that KIF5C is a direct target of miR-203, and overexpression of KIF5C partially offsets the tumor inhibitory effect of miR-203 on esophageal squamous cell carcinoma cells. In addition, miR-146, miR-196, miR-499, and others are all involved in biological behavior changes in esophageal cancer^25,26^.

After neoadjuvant immunotherapy, we first performed whole-transcriptome sequencing of esophageal squamous cell carcinoma and paracancerous tissues. Through bioinformatics analyses and in-vitro experiments, we explored the mechanism of mRNA–miRNA–lncRNA interactions in inhibiting esophageal squamous cell carcinoma by neoadjuvant immunotherapy and identified new and more accurate therapeutic targets and prognostic biomarkers. This approach provides new perspectives for research on immunotherapy for esophageal squamous cell carcinoma and provides a foundation for treating patients with esophageal squamous cell carcinoma.

## Results

### RNA sequencing

The whole-transcriptome sequencing of ten samples yielded a total CleanData of 148.48 G. Each sample’s effective data volume was distributed between 14.27 and 15.49 G, Q30 bases were distributed between 94.03 and 94.31%, and the total GC content was 49.49%. Alignment between the gene group of each sample was obtained by mapping the reads onto the reference gene group; the alignment rate was 97.32 to 98.21%. We identified 34,551 lncRNAs with a total length of 62,768,121 nucleotides (nt) and a mean length of 1,816.68 nt. In addition, 20,540 new circular RNAs were predicted, with a total length of 55,973,203 nt and a mean length of 2,725.08 nt. The gene group alignment rate was 92.81 to 96.25%, and the known miRNA alignment rate was 50.93 to 58.41%. The total number of samples mapped to known miRNAs was 1791. In total, 20,003 mRNAs were identified.

### Differential expression analysis

**Figure 1:**
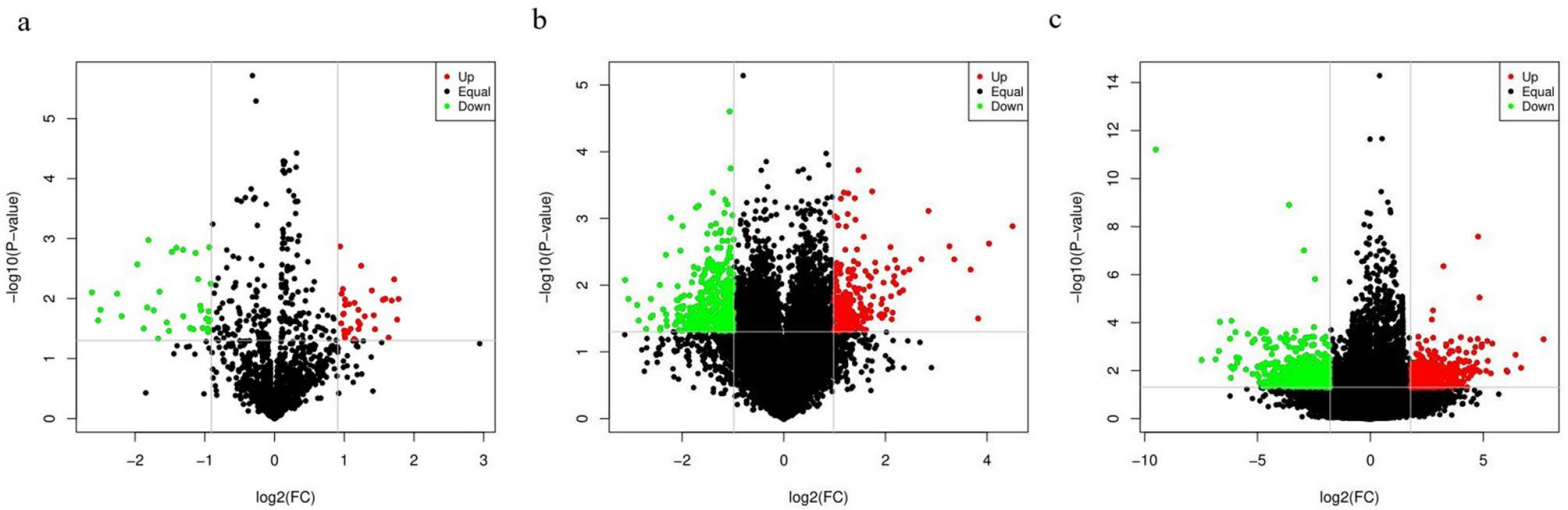
Volcano plots of differentially expressed genes. (**a**) Volcano plot of DEmiRNAs. (**b**) Volcano plot of DEmRNAs. (**c**) Volcano plot of DElncRNAs.

Differential gene analysis was applied to 20,003 mRNAs, 1,791 miRNAs, and 34,551 lncRNAs from ten samples after neoadjuvant immunotherapy using the limma package, and a volcano plot of differential gene expression was created. The differential gene analysis results are shown in Table 1, and a volcano map of differential gene expression is shown in Fig. 1.

**Table 1:** Identification of differential gene analysis.

	Differentially expressed genes	Upregulated genes	Downregulated genes	logFC >	P <
mRNA	584	219	365	0.98	0.05
miRNA	67	33	34	0.96	0.05
lncRNA	1047	539	508	1.78	0.05

### Differential gene set enrichment analysis (DEmRNAs and DEmiRNAs)

**Figure 2:**
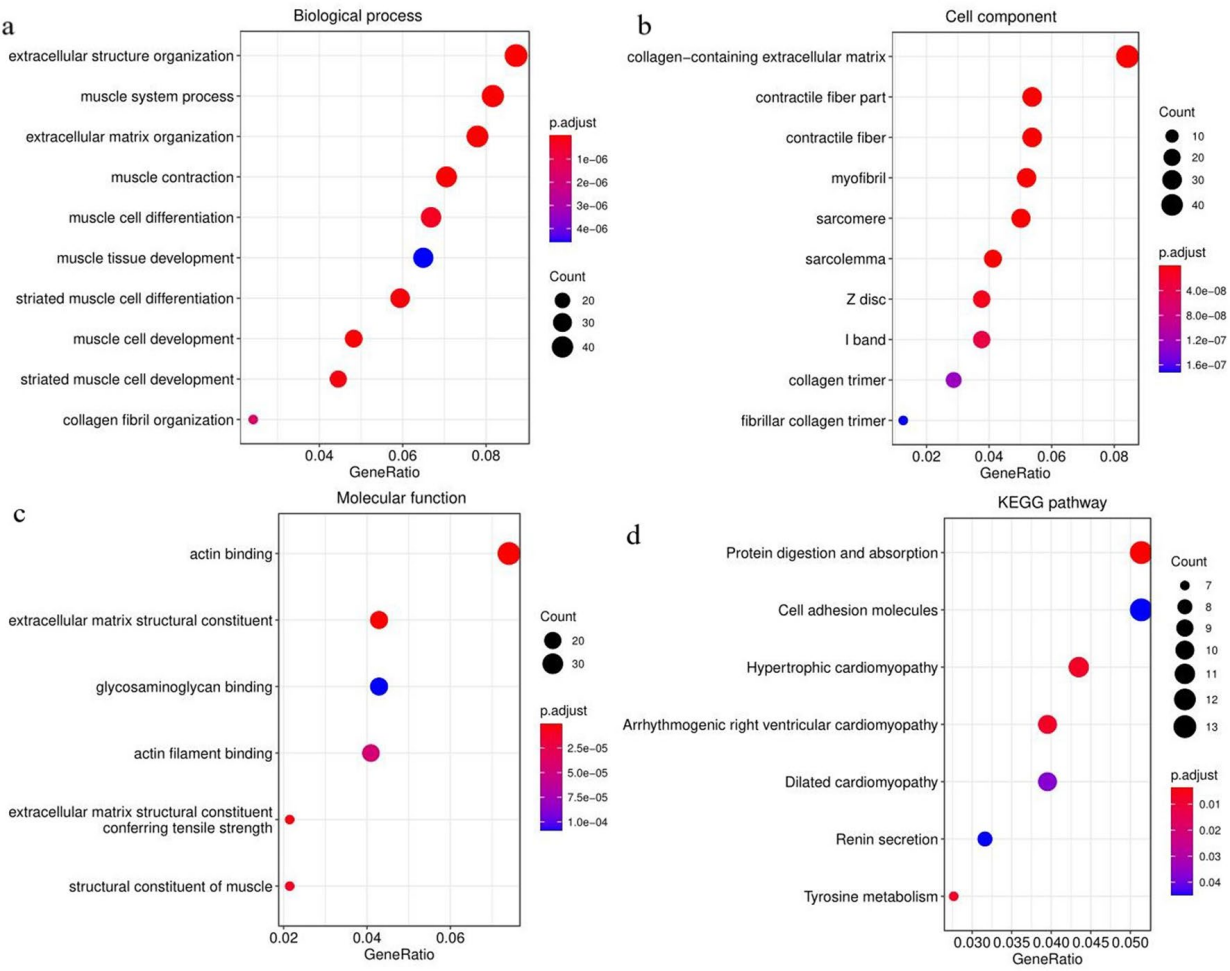
Bubble chart of the enrichment analyses of the differentially expressed genes. (**a**) GO enrichment analysis of biological processes. (**b**) GO enrichment analysis of cellular components. (**c**) GO enrichment analysis of molecular functions. (**d**) KEGG pathway analysis. P.adjust is the adjusted *p*-value.

As shown in the GO enrichment analysis (Fig. 2), 584 DEmRNAs of esophageal squamous cell carcinoma mainly participated in biological processes, such as extracellular structural organization, extracellular matrix organization, myoblast differentiation and development, and collagen fiber organization. Their cellular components mainly included collagen-containing extracellular matrix, contractile fibers, myogenic fibers, collagen trimers, and fibrillar collagen trimers. The molecular functions of these genes were significantly enriched for actin binding, extracellular matrix structural components, glycosaminoglycan binding, and muscle structural components. In addition, protein digestion and absorption, cell adhesion molecules, cardiomyopathy, renin secretion, and tyrosine metabolism pathways were significantly enriched in the KEGG pathway.

DEmiRNAs were mainly enriched in osteoclast differentiation, adhesive junctions, peptidyl threonine phosphorylation, and the epidermal growth factor receptor, insulin receptor, exogenous apoptotic, and phosphatidylinositol 3-kinase signaling pathways.

### Intergroup correlation analysis and total expression analysis of DEmiRNAs, DEmRNAs, and DElncRNAs

**Figure 3:**
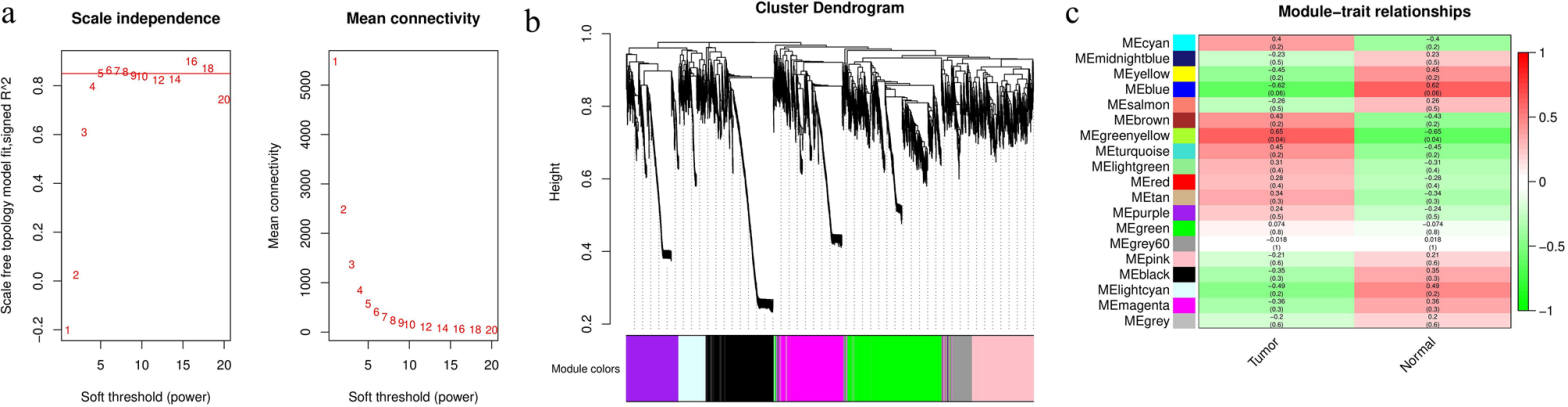
Identification of modules associated with the clinical information of miRNAs and lncRNAs. (**a**) Analysis of the scale-free fit index and mean connectivity for soft-thresholding power (β). (**b**) Cluster dendrogram of co-expression network modules ordered by the hierarchical clustering of genes based on the 1-topological overlap matrix (TOM) matrix. (**c**) Module-trait relationships. Each row corresponds to a color module, and each column corresponds to a clinical trait (cancer and normal).

Based on the principles of action of miRNA, mRNA, miRNA, and lncRNA, 7,279 miRNA–mRNA and 3,465 miRNA–lncRNA negative regulation relationship pairs were selected, respectively, and based on the principles of action of mRNA and lncRNA, 134,925 miRNA–lncRNA positive regulation relationship pairs were selected. To mine the set of mRNA and lncRNA genes with similar expression patterns associated with esophageal squamous carcinoma tumors after adjuvant immunotherapy, the total expression network of esophageal squamous cell carcinoma mRNA and lncRNA was constructed using the R package WGCNA. The 25th percentile of genes, based on the expression difference (17,832), was selected for WGCNA. To ensure a scale-free network, a power of β = 5 was chosen as the soft threshold parameter (Fig. 3a), and 18 gene co-expression modules were identified (Fig. 3b, each was assigned a different color). Heat maps of module-characteristic relationships were drawn to assess the association between each module and esophageal squamous carcinoma tumors (Fig. 3c). The results showed that the greenish-yellow module had the strongest association with esophageal squamous carcinoma tumors (r = 0.65, p = 0.04). The greenish-yellow module included 580 expression genes, including 129 mRNAs and 451 lncRNAs.

### Construction of ceRNA

**Figure 4:**
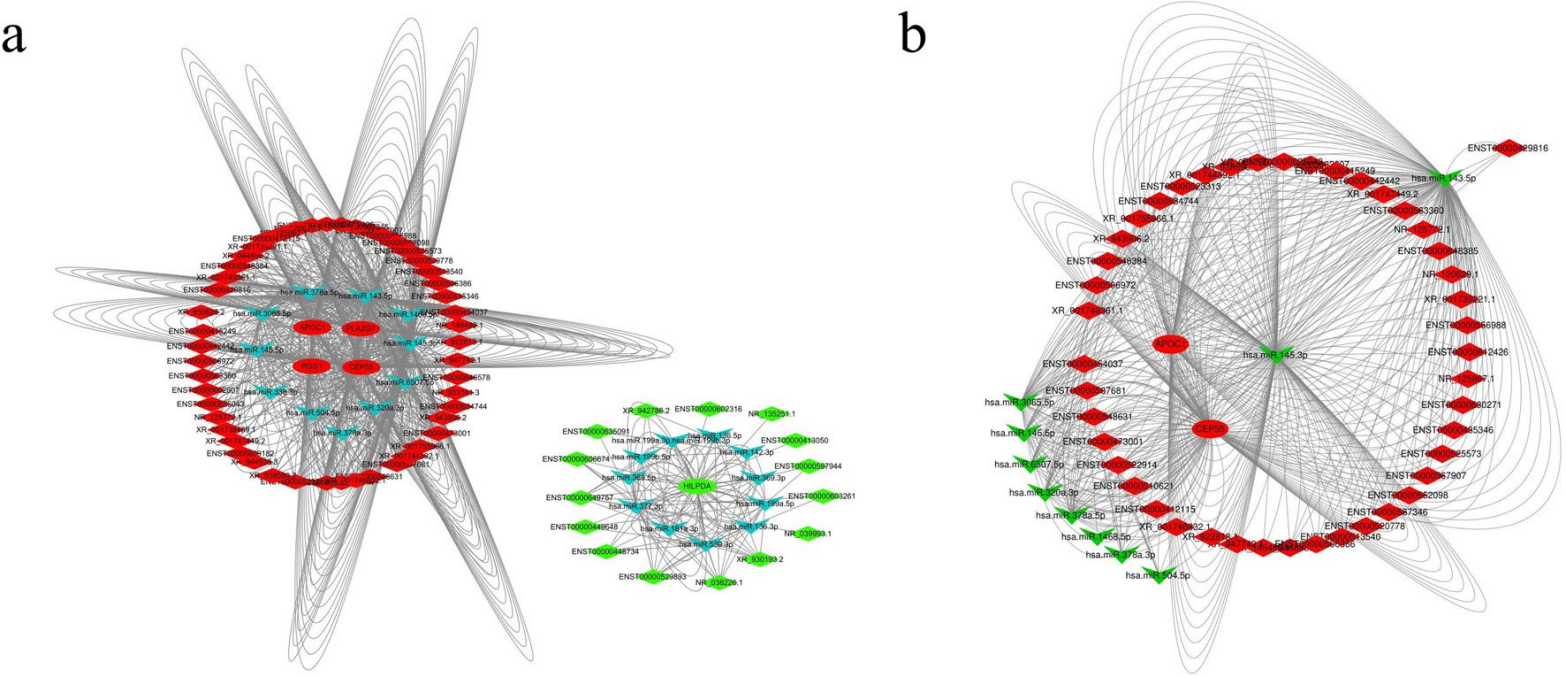
(**a**) ceRNA networks. (**b**) ceRNA networks related to prognosis (the ellipse nodes represent mRNA, arrowhead nodes represent miRNA, diamond nodes represent lncRNA, and red and green represent high and low expression, respectively).

The mRNA–lncRNA relationship pairs obtained using the Pearson correlation were intersected with the genes in the mRNA–lncRNA co-expression module obtained by the WGCNA method, and five mRNAs corresponding to the 116 lncRNAs were obtained as Pearson–WGCNA relationship pairs; five of these mRNAs were intersected with the obtained negative Pearson correlation relationship pairs of miRNA–mRNAs to obtain 29 miRNA–mRNA pairs; 116 of these lncRNAs were intersected with the obtained negative Pearson correlation relationship pairs of miRNA–lncRNAs to obtain 258 miRNA–lncRNA pairs. Based on the mined total expression data, the ceRNA (mRNA–miRNA–lncRNA) network was constructed and visualized using the Cytoscape (ver. 3.7.2) software (Fig. 4a). The constructed ceRNAs included five mRNAs (APOC1, PLA2G7, RGS1, CEP55, and HILPDA), 22 miRNAs, and 65 lncRNAs.

### Prognosis analysis and expression validation

**Figure 5:**
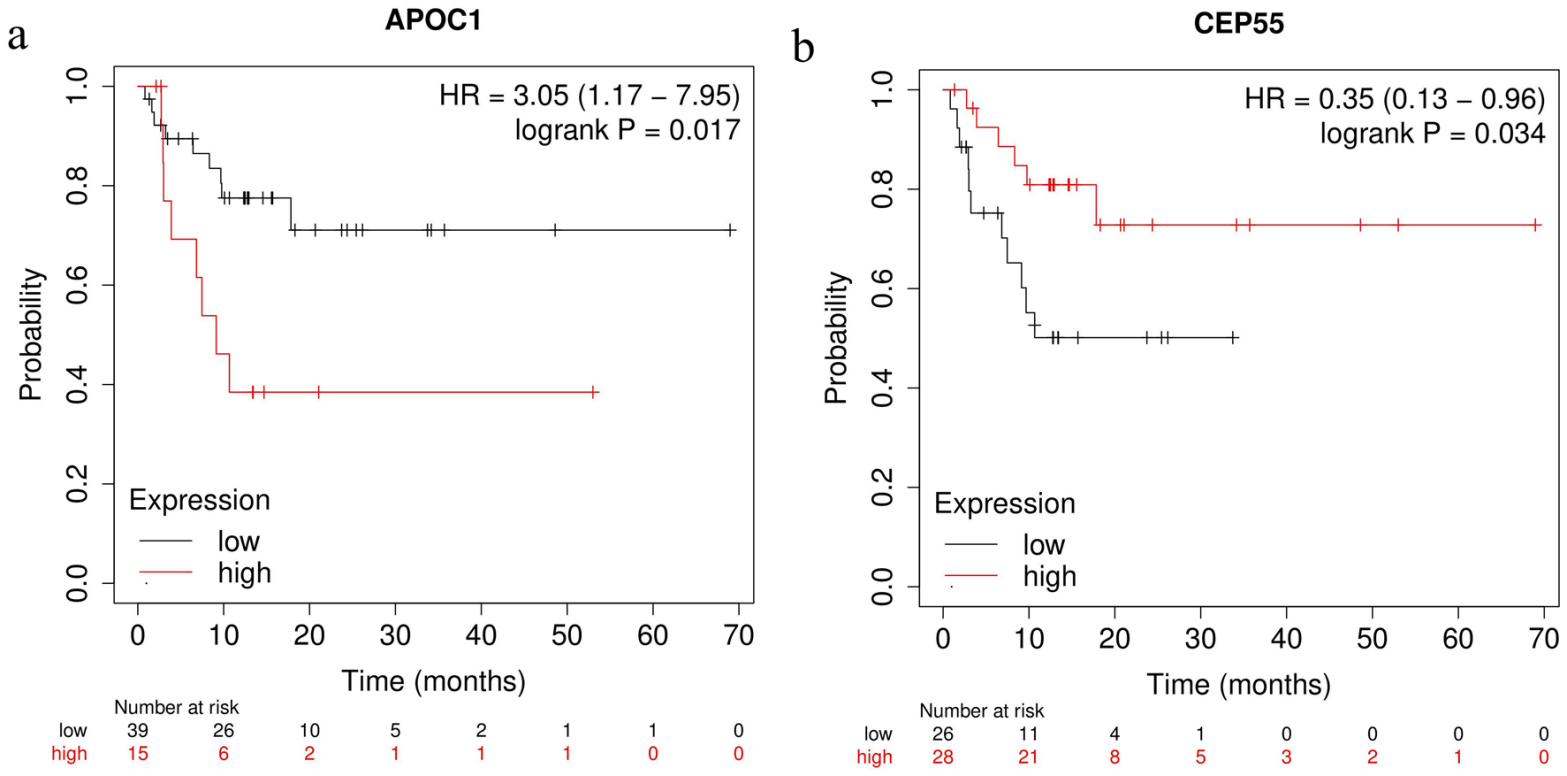
Correlation analysis of the association between hub mRNAs and recurrence-free survival. (**a**) *APOC1*. (**b**) *CEP55.*

APOC1 and CEP55 levels and recurrence-free survival of patients were correlated based on a KM plotter study (Fig. 5).

**Figure 6:**
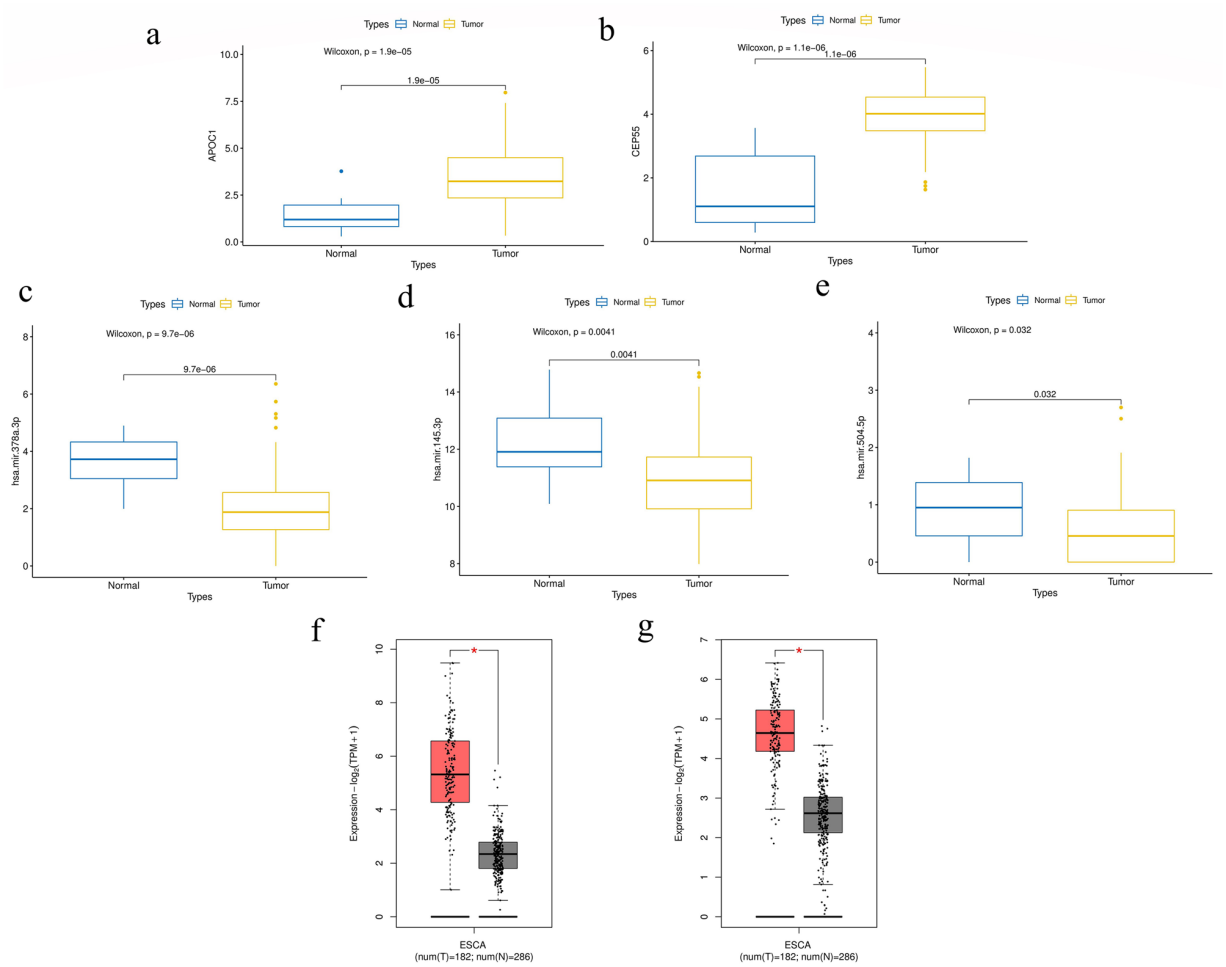
Validation of hub gene expression levels in esophageal squamous cell cancer. (**a**) Gene expression levels of *APOC1* in TCGA samples. (**b**) Gene expression levels of *CEP55* in TCGA samples. (**c**) Gene expression levels of hsa-miR-378a in TCGA samples. (**d**) Gene expression levels of hsa-miR-145 in TCGA samples. (**e**) Gene expression levels of hsa-miR-504 in TCGA samples. (**f**) Gene expression levels of *APOC1* in the GEPIA database. (**g**) Gene expression levels of *CEP55* in the GEPIA database.

Based on results described previously, the esophageal squamous cell carcinoma gene expression profile was downloaded from the TCGA database, including 60,483 mRNAs from 161 tumor samples and 11 normal samples and 1,881 miRNAs from 185 tumor samples and 12 normal samples. These were used to validate the expression levels of APOC1 and CEP55 and their related miRNAs (hsa-miR-143-5p, hsa-miR-145-3p, hsa-miR-378a-5p, hsa-miR-145-5p, hsa-miR-1468-5p, hsa-miR-3065-5p, hsa-miR-320a-3p, hsa-miR-378a-3p, hsa-miR-504-5p, and hsa-miR-6507-5p) in patients with esophageal squamous cell carcinoma. The results showed that APOC1, CEP55, hsa-miR-378a, hsa-miR-145, and hsa-miR-504 were significantly differentially expressed in tumor tissues compared to normal tissues (Fig. 6a–e).

To further validate the above results, we uploaded the key genes APOC1 and CEP55 to the Gene Expression Profiling Interactive Analysis (GEPIA) database for online analysis. The analysis showed that APOC1 and CEP55 were significantly upregulated in esophageal squamous cell carcinoma tissues, which was consistent with our analysis results (Fig. 6f, g).

Key mRNA–miRNA–lncRNA among the ceRNAs was further visualized using the Cytoscape software (Fig. 4b).

### RT-PCR validation

Expression levels were normalized to those of APOC1, CEP55, and miRNAs (hsa-miR-143-5p, hsa-miR-145-3p, hsa-miR-378a-5p, hsa-miR-145-5p, hsa-miR-1468-5p, hsa-miR-3065-5p, hsa-miR-320a-3p, hsa-miR-378a-3p, hsa-miR-504-5p, and anhashsa-miR-6507-5p) and calculated using the 2-ΔΔCt method^27^.

**Figure 7:**
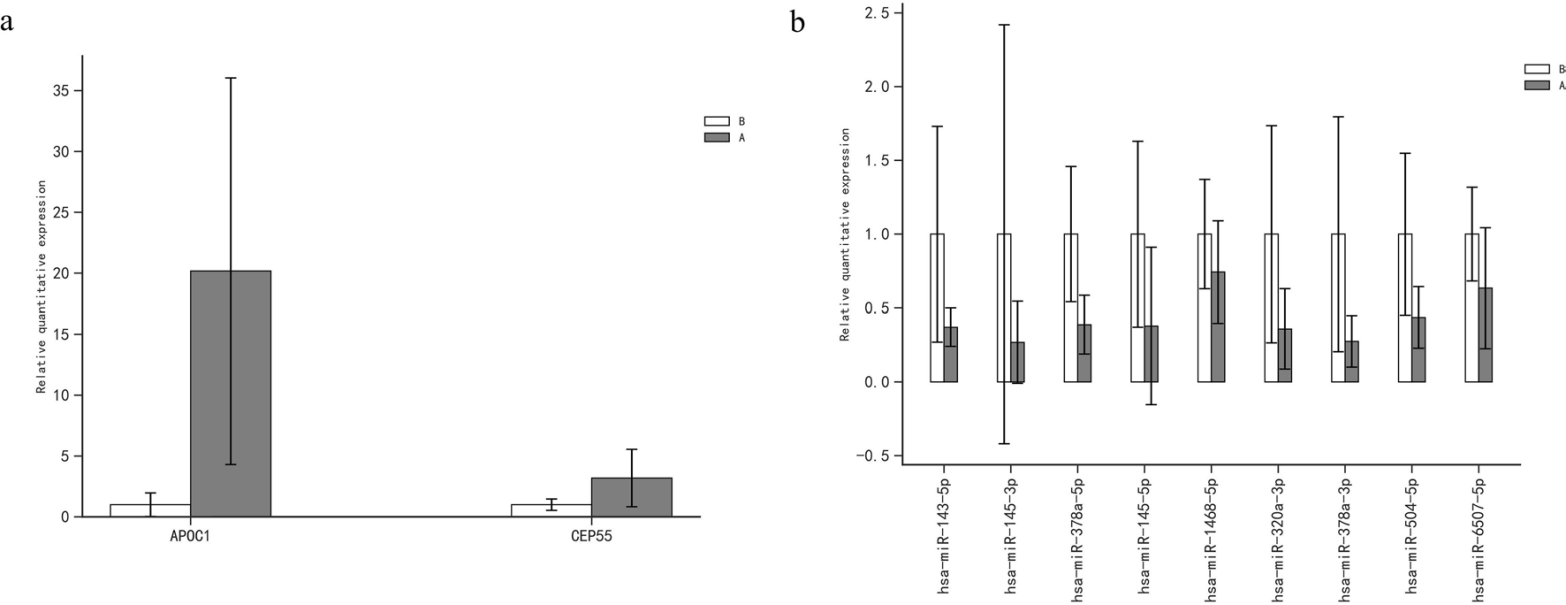
Column chart of RT-PCR validation (A and B represent esophageal squamous cell cancer and normal tissues, respectively). (**a**) Expression levels of mRNAs in esophageal squamous cell cancer and normal tissues. (**b**) Expression levels of miRNAs in esophageal squamous cell cancer and normal tissues.

Validation using RT-PCR showed that APOC1 expression was significantly higher in esophageal squamous carcinoma tissues than in paraneoplastic tissues after neoadjuvant immunotherapy (20.174 ± 15.870 vs. 1 ± 0.967, p < 0.001); CEP55 expression was significantly higher in esophageal squamous carcinoma tissues than in paraneoplastic tissues (3.190 ± 2.361 vs. 1 ± 0.467, p < 0.001); moreover, we further validated the expression levels of predicted mihass (hsa-miR-143-5p, hsa-miR-145-3p, hsa-miR-378a-5p, hsa-miR-145-5p, hsa-miR-1468-5p, hsa-miR-3065-5p, hsa-miR-320a-3p, hsa-miR-378a-3p, hsa-miR-504-5p, and hsa-miR-6507-5p) in patients with esophageal squamous carcinoma using RT-PCR, which showed that hsa-miR-143-5p (0.370 ± 0.129 vs. 1 ± 0.731, p = 0.032), hsa-miR-378a-5p (0.387 ± 0.198 vs. 1 ± 0.458, p = 0.016), hsa-miR-320a-3p (0.358 ± 0.271 vs. 1 ± 0.735, p = 0.042), and hsa-miR-378a-3p (0.275 ± 0.174 vs. 1 ± 0.796, p = 0.028) had low expression in esophageal squamous carcinoma, which statistically differed from that in the control (Fig. 7).

### Correlation between the expression of key genes and immune infiltration

**Figure 8:**
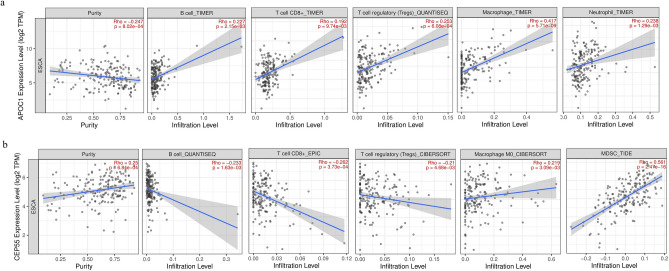
Relationship between the key genes and immune cell infiltration. (**a**) *APOC1*. (**b**) *CEP55*.

Immune infiltration analysis was performed on the two key genes using the TIMER immune infiltration database. The results demonstrated that the expression of APOC1 and CEP55 and the purity of esophageal squamous cell carcinoma cells were correlated (p < 0.05). In addition, APOC1 expression positively correlated with the degree of infiltration of B cells, CD8 + T cells, regulatory T cells (TREGS), macrophages, and neutrophils (p < 0.05; Fig. 8a). CEP55 expression was negatively correlated with B cells, CD8 + T cells, and regulatory T cells but positively correlated with bone marrow-derived suppressor cells and macrophages (p < 0.05; Fig. 8b).

## Discussion

In this study, we found numerous dysregulated RNAs in esophageal squamous cell carcinoma samples after neoadjuvant immunotherapy using deep sequencing and high-throughput screening. In total, 584 DEmRNAs, 67 DEmiRNAs, and 1,047 DElncRNAs were identified by differential gene analysis. Based on this, miRNA–mRNA, miRNA–lncRNA, and mRNA–lncRNA relationship pairs were obtained using the Pearson correlation method, and based on the mRNA–lncRNA co-expression module obtained by the WGCNA method, a ceRNA (mRNA–miRNA–lncRNA) network closely related to esophageal squamous carcinoma was constructed. Two key mRNAs related to esophageal squamous cell carcinoma, APOC1 and CEP55, and miRNAs closely associated with them, including hsa-miR-378a, hsa-miR-145, and hsa-miR-504, were identified using expression and survival analyses. These results were also validated using related data downloaded from the TCGA public database. Subsequently, when we performed PCR validation on surgical specimens after neoadjuvant immunotherapy, we found that APOC1 and CEP55 were significantly upregulated and hsa-miR-378a-5p and hsa-miR-378a-3p were considerably downregulated in esophageal squamous cell carcinoma samples. Immune infiltration analysis demonstrated that the expression of APOC1 and CEP55, the purity of esophageal squamous cell carcinoma cells, and numerous immune infiltration cells were closely related.

Chen et al.^28^ found that APOE, APOC1, and SPP1 were highly expressed in esophageal squamous carcinoma tumor-associated macrophages when single-cell transcriptome sequencing was performed in patients with esophageal squamous carcinoma treated with paclitaxel in combination with platinum-based chemotherapy. It has been suggested that APOC1 is co-expressed with immune checkpoints, MHC molecules, and other immune-related genes in tumors. Therefore, it can induce tumor invasion and metastasis by affecting macrophage secretion^29^. In this study, we found that APOC1 was also highly expressed in cancer tissues after neoadjuvant immunotherapy for esophageal squamous cell carcinoma, which is consistent with other studies. More importantly, we speculate that APOC1 might better predict the prognosis after neoadjuvant immunotherapy by regulating the immune microenvironment by APOC1. We will focus on this aspect in future studies.

We found that CEP55 plays an essential role in cytokinesis; it has also been reported to promote the contraction of intracellular bridges to promote cytokinesis^30^. Overexpression of CEP55 was correlated with poor prognosis of esophageal squamous cell carcinoma^31,32^. Another study proposed that CEP55 promotes the proliferation, migration, and invasion of esophageal squamous cell carcinoma via the PI3K/Akt pathway^33^. We observed overexpression of CEP55 in esophageal cancer tissues after neoadjuvant immunotherapy. Some researchers reported that CEP55 can be used as a predictive indicator for immunotherapy in adrenocortical carcinoma^34^. Lu et al.^35^ found that CEP55 was highly expressed in pancreatic ductal adenocarcinoma cells and that its expression in pancreatic patients receiving immunotherapy could reflect the efficacy and prognosis of immunotherapy. Therefore, CEP55 can be used as a predictive indicator of the efficacy of neoadjuvant tumor immunotherapy^35^. Thus, CEP55 could be another decent target for efficacy prediction after neoadjuvant immunotherapy of esophageal squamous cell carcinoma. We will further explore this aspect in future studies.

miR-378a is derived from the peroxisome proliferator-activated receptor gamma coactivator 1-β gene. It has two mature structures: miR-378a-3p and miR-378a-5p^36^. miR-378a participates in numerous biological processes^37–39^ and inhibits the proliferation, migration, and invasion of esophageal squamous cell carcinoma cells^40^. SLC2A1-AS1 may be important in driving esophageal squamous carcinoma tumorigenesis, progression, and glycolysis through the SLC2A1-AS1/miR-378a-3p/Glut1 signaling axis^41^. Another study demonstrated that as a ceRNA, LINC00514 indirectly upregulates the expression of SPHK1 to promote the progression of esophageal squamous cell carcinoma by absorbing miR-378a-5p^42^.

In this study, we intended to investigate the precise mechanism of the progression of esophageal squamous cell carcinoma after neoadjuvant immunotherapy from an mRNA–miRNA–lncRNA network perspective. By combining expression analysis, survival analysis, and wet and dry research validation, a key pathway (hsa-miR-378-5p-APOC1/CEP55) of esophageal squamous cell carcinoma progression after neoadjuvant immunotherapy was identified. This pathway demonstrates that miR-378a-5p acts as an active tumor suppressor by targeting and regulating APOC1 and CEP55 during the onset and progression of esophageal squamous cell carcinoma.

Neoadjuvant immunotherapy has been a cross-generational treatment method for esophageal squamous cell carcinoma since the advent of neoadjuvant chemotherapy and neoadjuvant radiotherapy. While some patients with esophageal squamous cell carcinoma responded with complete remission, treatment had mediocre efficacy for many other patients. To date, we have only tried to select targets that may benefit from neoadjuvant immunotherapy at the clinical level, and in-depth molecular biological mechanisms still need to be discovered, which is critical for efficient treatment of esophageal squamous cell carcinoma.

Despite the small sample size of this study, we identified some molecular mechanisms that may explain neoadjuvant immunotherapy for esophageal squamous cell carcinoma; however, the study had a few limitations. First, this study was only focused on transcriptome sequencing analysis of cancer and noncancerous adjacent tumor tissues from patients with esophageal squamous cell carcinoma who underwent immunotherapy. Therefore, further studies related to differential gene expression before and after immunotherapy for esophageal squamous cell carcinoma are warranted. Second, since tumor samples are heterogeneous, the patients included in this study might have only been representative of some patients with esophageal squamous cell carcinoma. Third, most patients underwent endoscopy before neoadjuvant immunotherapy and had insufficient specimens for whole-transcriptome sequencing. Finally, bioinformatic analysis demonstrates that hsa-miR-378-5p could simultaneously regulate APOC1 and CEP55; however, key gene interactions require validation by more basic and in-depth experiments. However, in this study, we conducted a comprehensive analysis of the tumor and paracancerous specimens of patients with esophageal squamous cell carcinoma who had undergone adjuvant immunotherapy and laid the foundation for further research on the therapeutic targets of esophageal squamous cell carcinoma.

## Materials and methods

### Patients and specimens

Our study included patients diagnosed with ESCC. All patients gave their informed consent to participate in the study. Ethics approval was provided by the Ethics Committee of the Affiliated Hospital of Inner Mongolia Medical University reference number KY202210. All methods were performed in accordance with the Declaration of Helsinki.

We collected cancer and paracancer specimens from five patients with esophageal squamous cell carcinoma to determine ceRNA differences between cancer and paracancer after immunotherapy. Among the five patients enrolled, intravenous infusion of 200 mg of carrilizumab was administered for 30 min every three weeks. Combined with cisplatin and albumin-paclitaxel (the specific dosage was adapted according to the actual situation of the patient and the drug instructions) treatment, this was a 21-day cycle. After two consecutive cycles, the patients underwent surgery after four further weeks. Inclusion criteria: tissues of patients with histologically confirmed middle and advanced esophageal squamous cell carcinoma (cT1b-cT2N + or cT3-cT4a, any N– suspected involvement of surrounding organs but no clear cT4b) with complete imaging examination and preoperative immunoneoadjuvant therapy were considered resectable by thoracic surgeons; the age of the patients was 18–75 years (male and female); and patients have not received any systematic anti-tumor therapy, including surgery, chemotherapy, radiotherapy, targeting, and immunotherapy. Exclusion criteria: patients who had not used immunotherapy before surgery; postoperative pathological returns showed complete pathological response or no immunotherapy response; a history of other malignancies; and patients who did not consent to the clinical research.

### RNA extraction

Total RNA was extracted from tissues after immunotherapy for esophageal squamous cell carcinoma using the Eastep® Super Total RNA Extraction Kit according to the manufacturer’s specifications (Shanghai Plommedical Biological Products; Catalog number: LS1040). The yield of RNA was determined using a NanoDrop 2000 spectrophotometer (Thermo Scientific, Waltham, MA, USA), and its integrity was evaluated using agarose gel electrophoresis stained with ethidium bromide.

### Analysis of differentially expressed genes

Based on RNA sequencing data, differentially expressed genes between the diseased and control groups (DEmiRNAs, DEmRNAs, and DElncRNAs) were screened using the R^43^ package limma ver. 3.42.2^44^. The screening thresholds were set to |log fold change (FC)|> [mean(|logFC|) + 2SD(|logFC|)], with a *p*-value < 0.05. The package ggplot2 version 3.3.3^45^ was used to create volcano plots of differential genes.

### Enrichment analysis of differential genes (DEmRNAs and DEmiRNAs)

To analyze the biological processes involved in the onset of esophageal squamous cell carcinoma, functional enrichment analysis (GO) and signaling pathway analysis using the Kyoto Encyclopedia of Genes and Genomes (KEGG) were performed on the screened DEmRNAs using the profiler package ver. 3.14.3. The package ggplot2 was used to create the enrichment bubble chart. GO and signaling pathway analysis (KEGG) were performed on the screened DEmRNAs using the online tool miEAA (https://ccb-compute2.cs.uni-saarland.de/mieaa2/).

### Intergroup correlation analysis and co-expression analysis of differential genes (DEmiRNAs, DEmRNAs, and DElncRNAs)

Based on the expression levels of differentially expressed genes (DEmiRNAs, DEmRNAs, and DElncRNAs) in the sample, intergroup correlations between DEmiRNAs and DEmRNAs, and DEmiRNAs and DElncRNAs were analyzed using Pearson’s product difference correlation coefficient. Pairwise deletion was applied to the missing data. The correlation analysis set the threshold to have the absolute value of a correlation coefficient higher than 0.60 and a *p*-value < 0.05. Since miRNA and target mRNA are not complementary, miRNA can inhibit target gene expression at the protein translation level. At the same time, numerous lncRNAs have structures similar to that of mRNA. Therefore, miRNAs can negatively regulate lncRNAs through a mechanism similar to that acting on mRNAs^46^. The negative regulatory relationship pairs miRNA–mRNA and miRNA–lncRNA were selected to further screen the target genes of miRNA.

Based on the principles of action of mRNA and lncRNA, Pearson’s intergroup correlation of mRNA and lncRNA was analyzed. The correlation analysis set the threshold to have the absolute value of a correlation coefficient greater than 0.60 and a *p*-value < 0.05. A positive regulatory relationship pair, mRNA–lncRNA, was selected. At the same time, the co-expression network of esophageal squamous cell carcinoma mRNA and lncRNA was constructed using weighted correlation network analysis in R (WGCNA ver. 1.69)^47,48^. The 25th percentile of genes based on the expression difference (17,832) was selected for weighted gene co-expression network analysis. A co-expression network module of esophageal squamous cell carcinoma mRNA and lncRNA was constructed.

### Construction of ceRNA

Based on the miRNA–mRNA, miRNA–lncRNA, and mRNA–lncRNA relationship pairs obtained by the Pearson correlation method and the mRNA–lncRNA co-expression module obtained using the WGCNA method, a ceRNA (mRNA–miRNA–lncRNA) network closely related to esophageal squamous carcinoma was constructed. Cytoscape (ver. 3.7.2) was used to visualize the constructed Interworking Network.

### Prognosis analysis and expression validation

To investigate the time elapsed between achieving a complete remission until the appearance of recurrence in patients with esophageal squamous cancer after neoadjuvant immunotherapy, the online tool KM plotter (http://kmplot.com/analysis/) was used to explore the relationship between key mRNAs in ceRNA and patients’ recurrence-free survival (RFS). Esophageal squamous cell carcinoma was selected for the type of cancer, median for patient classification, and RFS for the survival status. Additionally, key mRNAs related to survival were selected.

To further validate the reliability of the key genes, esophageal squamous cell carcinoma mRNA and miRNA expression data were downloaded from the TCGA database. The differences in the expression levels of key mRNAs and their related miRNAs in tumors and normal tissues were validated, and boxplots were created.

The GEPIA database (http://gepia.cancer-pku.cn/) was used to analyze the differences in expression between tumor and normal tissues. For example, the expression of key genes in esophageal squamous cell carcinoma and normal tissues was validated using the GEPIA database.

Validated key mRNA–miRNA–lncRNA among the ceRNA were further visualized using the Cytoscape software.

### Real-time quantitative reverse transcription (RT)-polymerase chain reaction (PCR)

Quantification was performed using a two-step reaction: RT and PCR. Each RT reaction consisted of 0.5 μg of RNA, 2 μL of 5 × TransScript All-in-one SuperMix for qPCR, and 0.5 μL of gDNA Remover, in a total volume of 10 μL. The reactions were performed in a GeneAmp® PCR System 9700 (Applied Biosystems, Foster City, CA, USA) for 15 min at 42 °C and 5 s at 85 °C. Subsequently, 10 μL of RT reaction mix was diluted ten times in nuclease-free water and kept at -20 °C.

Real-time PCR was performed using LightCycler® 480 II Real-time PCR Instrument (Roche, Basel, Switzerland) with a 10-μL PCR mixture that included 1 μL of cDNA, 5 μL of 2 × PerfectStart™ Green qPCR SuperMix, 0.2 μL of forward primer, 0.2 μL of reverse primer, and 3.6 μL of nuclease-free water. The reactions were incubated in a 384-well optical plate (Roche, Basel, Switzerland) at 94 °C for 30 s, followed by 45 cycles at 94 °C for 5 s, and 60 °C for 30 s. Each sample was analyzed in triplicate. At the end of the PCR, a melting curve analysis was performed to validate the specific generation of the expected PCR product. Primer sequences were designed in the laboratory and synthesized by TsingKe Biotech (Beijing, China) based on the mRNA sequences obtained from the National Center for Biotechnology Information database.

### Immune infiltration analysis

Cancer immune infiltrate database Tumor IMmune Estimation Resource (TIMER) 2.0 (http://timer.cistrome.org/)^49^ was used to analyze the relationship between the expression of key pathogenic genes and the extent of immune cell infiltration. Specifically, these included B cells, CD8 + T cells, regulatory T cells, macrophages, bone marrow-derived suppressor cells, and neutrophils.

## Data Availability

All generated sequencing data for analysis during this study are uploaded the relevant data to the Github public platform at https://github.com/jiarong0719/RNA-data.
